# Effect of Rearing System on the Straight and Branched Fatty Acids of Goat Milk and Meat of Suckling Kids

**DOI:** 10.3390/foods9040471

**Published:** 2020-04-09

**Authors:** Guillermo Ripoll, María Jesús Alcalde, Anastasio Argüello, María de Guía Córdoba, Begoña Panea

**Affiliations:** 1 Instituto Agroalimentario de Aragón–IA2–(CITA-Universidad de Zaragoza), C/Miguel Servet, 177, 50013 Zaragoza, Spain; bpanea@aragon.es; 2 Animal Production and Health Department, Centro de Investigación y Tecnología Agroalimentaria de Aragón, Avda. Montañana, 930, 50059 Zaragoza, Spain; 3 Department of Agroforesty Science, Universidad de Sevilla, Crta. Utrera, 41013 Sevilla, Spain; aldea@us.es; 4 Department of Animal Pathology, Animal Production and Science and Technology of Foods, Universidad de Las Palmas de Gran Canaria, 35416 Las Palmas, Spain; tacho@ulpgc.es; 5 Nutrición y Bromatología, Instituto Universitario de Investigación de Recursos Agrarios (INURA), Nutrición y Bromatología, Escuela de Ingeniería Agrarias, Universidad de Extremadura, Avda. Adolfo Suarez s/n, 06007 Badajoz, Spain; mdeguia@unex.es

**Keywords:** goat, milk, BCFA, replacer, methyl, colostrum

## Abstract

Goat meat is considered healthy because it has fewer calories and fat than meat from other traditional meat species. It is also rich in branched chain fatty acids that have health advantages when consumed. We studied the effects of maternal milk and milk replacers fed to suckling kids of four breeds on the straight and branched fatty acid compositions of their muscle. In addition, the proximal and fatty acid compositions of colostrum and milk were studied. Goat colostrum had more protein and fat and less lactose than milk. Goat milk is an important source of healthy fatty acids such as C18:1 c9 and C18:2 n–6. Suckling kid meat was also an important source of C18:1c9. Dairy goat breeds had higher percentages of *trans* monounsaturated fatty acids (MUFAs) and most of the C18:1 isomers but lower amounts of total MUFAs than meat breeds. However, these dairy kids had meat with a lower percentage of conjugated linoleic acid (CLA) than meat kids. The meat of kids fed natural milk had higher amounts of CLA and branched chain fatty acids (BCFAs) and lower amounts of n–6 fatty acids than kids fed milk replacers. Both milk and meat are a source of linoleic, α-linolenic, docosahexaenoic, eicosapentaenoic and arachidonic fatty acids, which are essential fatty acids and healthy long-chain fatty acids.

## 1. Introduction

Approximately 119,000 tons of caprine meat were produced in Europe in 2017 [1]. However, Mediterranean goat farms are mainly focused on dairy production, including cheese and milk products, because they have higher prices than cow milk [2]. In addition, goat milk is also generating great interest for human consumption due to its nutritional advantages and lack of allergenicity compared to cow milk [3]. Accordingly, Europe produces 2,824,715 tons of goat milk, and 45% is produced in South Europe [1]. Although most income per goat on the dairy farm comes from the sale of milk, 20% of the total income comes from the sale of kids [4]. These kids are weaned very early and reared with milk replacers. These milk replacers are specifically formulated for kids, resulting in good daily weight gain. Moreover, this meat is perceived by consumers to be high-quality meat, with most kids being slaughtered at the very light carcass weight of 5–7 kg [5]. However, some farmers, especially those who use autochthonous breeds, believe that early separation decreases both milk yield and growth of the kids. In addition, they also believe that meat from kids reared with milk replacers is tough [6] and, as a consequence, they are opposed to this practice. This belief could be explained because most of the kid meat with high pH, which may induce tough meat, comes from kids raised on milk replacers [7]. On the other hand, the meat of kids reared with milk replacers was preferred by consumers according to visual appraisal, and consequently, as the purchase intention of these consumers was high [8,9]. Another advantage of meat from suckling kids fed natural milk is that it has a high percentage of hexanal, which has been positively related to both the flavor and overall acceptability [10].

On the other hand, goat meat is considered healthy because it has fewer calories and fat than meat from other meat species, such as pork or lamb [11] and because its fatty acid profile complies with the recommendation of the World Health Organization, which states that trans fatty acid intake should not reach more than 5% [12]. In addition, goat meat is rich in branched chain fatty acids (BCFAs), and BCFAs are of interest for two main reasons: first, BCFAs, particularly short chain BCFAs, have an impact on the characteristic flavor of meat and dairy products [13,14,15,16]. Second, some authors have described health advantages with BCFA consumption. Additionally, the intake of BCFAs is related to the correct function of the newborn gut [17] and the induction of apoptosis in breast cancer cells [18]. Considering these benefits, BCFAs could be considered bioactive compounds and deserve deep insight. BCFAs are mainly saturated fatty acids with at least one methyl branch on the carbon chain. Often, the branch is close to the end of the chain, producing the *iso*- and *anteiso*- isomers when the methyl branch is on the penultimate or antepenultimate carbon atoms. BCFAs are synthetized by bacteria as a main component of the bacterial membrane. Hence, BCFAs are found in meat and milk from ruminants because of rumen bacterial activity. In general, adipose tissues of goats are richer in these fatty acids than those of other ruminants [19]. However, very light suckling kids are functionally non-ruminants [20], and the presence of BCFAs in their meat probably originates mainly from maternal milk. In addition, kids fed milk replacers from cow milk do not consume many BCFAs. Setting aside the origins of the BCFAs in meat, we studied the effects of feeding suckling kids with maternal milk or milk replacers on the straight and BCFAs of their muscles. In addition, the proximal and fatty acid compositions of colostrum and milk of four breeds were studied.

## 2. Materials and Methods

### 2.1. Animals

All procedures were conducted according to the guidelines of Directive 2010/63/EU on the protection of animals used for experimental and other scientific purposes [21].

One hundred and twenty-four suckling male kids of 4 goat breeds (Cabra del Guadarrama, GU; Palmera, PL; Retinta, RE; Tinerfeña, TI) were evenly reared at two (PA and TI) or three farms (GU and RE) per breed in their respective local areas. Therefore, 15, 15, 15 and 16 kids of GU, PL, RE and TI, respectively, were fed milk replacers (MR), and 16, 16, 15 and 16 kids of GU, PL, RE and TI, respectively, were fed natural milk (NM). Animals were selected to be as unrelated as possible to ensure that the full range of genetic diversity was present within the breeds used in the study. Animals were all born from a single parturition. Kids in the MR rearing systems were fed colostrum for the first two days and had free access to milk replacer 24 h a day, which was suckled from a teat connected to a unit for feeding a liquid diet. The goats grazed the natural resources of the area during the day and were supplemented with hay of the pastures and similar commercial concentrates between breeds.

### 2.2. Milk Sampling

Commercial milk replacers were reconstituted at 17% (w/v) and given warm (40 °C). The main ingredients were skimmed milk (≈ 60%) and whey. The chemical composition (on an as-dry matter basis) of milk replacers was as follows: total fat 25% ± 0.6, crude protein 24% ± 0.5, crude cellulose 0.1% ± 0.0, ash 7% ± 0.6, Ca 0.8% ± 0.1, Na 0.5% ± 0.2, P 0.7% ± 0.0, Fe 36 mg/kg ± 4.0, Cu 3 mg/kg ± 1.7, Zn 52 mg/kg ± 18.8, Mn 42 mg/kg ± 14.4, I 0.22 mg/kg ± 0.06, Se 0.1 mg/kg ± 0.06 and BHT 65 ppm ± 30. Kids in the NM rearing system suckled directly from dams with no additional feedstuff. At night, they were housed with their dams in a stable. Kids in both rearing systems had no access to concentrates, hay, forages or other supplements.

The natural milk of dams was collected from 10:00 h to 11:00 h in the morning at 1, 10 and 30 d of lactation. Two 50 mL Falcon tubes were filled with milk and three drops of Azidiol (Panreac Applichem, Barcelona, Spain). No oxytocin was used. The chemical composition (protein, fat and lactose) of the milk was determined by using a DMA2001 Milk Analyzer (Miris Inc., Uppsala, Sweden). A subsample of natural milk at 1 and 30 d of lactation was freeze-dried and stored at −80 °C until fatty acid analysis.

### 2.3. Carcass Sampling

The 124 kids were slaughtered at a live weight of 8.4 kg ± 0.12 kg. Standard commercial procedures according to the European normative of protection of animals at the time of killing [22] were followed. Head-only electrical stunning was applied (1.00 A) to the kids, which were then exsanguinated and dressed. Thereafter, the hot carcasses, including the heads and kidneys, were weighed to achieve a hot carcass weight (HCW) of 5.0 kg ± 0.10 kg. Afterwards, carcasses were hung by the Achilles tendon and chilled for 24 h at 4 °C. After carcass chilling, the *longissimus thoracis* muscle of the left half of the carcasses was extracted, vacuum packed and frozen at −20 °C until fatty acid composition analyses.

### 2.4. Fatty Acid Analyses

The fatty acid methyl esthers (FAMEs) from lyophilized milk fat samples were prepared by direct transesterification using KOH in methanol (2 N) and extracted with hexane [23]. Fat depots for fatty acid profile analysis were processed according to Folch et al. [24].

The determination of FAME was carried out using a Bruker 436 Scion gas chromatograph (Bruker, Billerica, MA, USA) equipped with a cyanopropyl capillary column (BR-2560, 100 m × 0.25 mm ID × 0.20 µm thickness, Bruker, Billerica, MA, USA), a flame ionization detector and Compass CDS software. Fatty acid quantification was performed as described in the UNE-EN 12966-4 Official Method (2015). The identification was performed using the GLC 538 and GLC 463 standard references (Nu-Chek-Prep Inc., Elysian, Minnesota, USA). Fatty acid contents are expressed as a percentage of the total amount of identified fatty acids. After individual fatty acid determinations, the total contents of saturated fatty acids (SFAs), monounsaturated fatty acids (MUFAs), polyunsaturated fatty acids (PUFAs), PUFA n–6 and PUFA n–3 were calculated. The PUFA n–6/n–3 ratios were also calculated. Desirable fatty acids of the meats were calculated as MUFA + PUFA + C18:0 [25]. In addition, the sums of conjugated linoleic acid (CLA), *iso*- and *anteiso*- BCFA, *cis*- and *trans*- MUFA were also calculated.

### 2.5. Statistical Analysis

All statistics were calculated using the XLSTAT statistical package v.3.05 (Addinsoft, New York, NY, USA). The proximal composition and fatty acid composition of milk were analyzed using the MIXED procedure for repeated measures. The factors included were breed as between-subject fixed effects, time as within-subject effects and random animal effects as subjects (experimental units). The lowest Akaike Information Criterion (AIC) was used to choose the matrix of the error structure. Least square means were estimated, and differences were tested with a t-test at the 0.05 level.

The fatty acid composition of meat was analyzed using the ANCOVA procedure with the breed (B) and the rearing system (RS) as fixed effects and the hot carcass weight (HCW) as a covariate. The HCW was used as covariate to avoid the influence of weight differences on the fatty acid composition. The least square means were adjusted for an HCW of 5.02 kg. Differences between means were tested with Duncan’s test at a 0.05 level of significance.

Two principal component analyses were performed with the main fatty acid groups of both milk and meat. The variables included in these principal component analyses were SFA, MUFA, PUFA, n–3, n–6, n–6:n–3 ratio, ΣCLA, Σ*iso*-BCFA, Σ*anteiso*-BCFA, ΣBCFA, *cis*-MUFA, and Σ*trans*-MUFA.

## 3. Results

### 3.1. Milk

**Figure 1:**
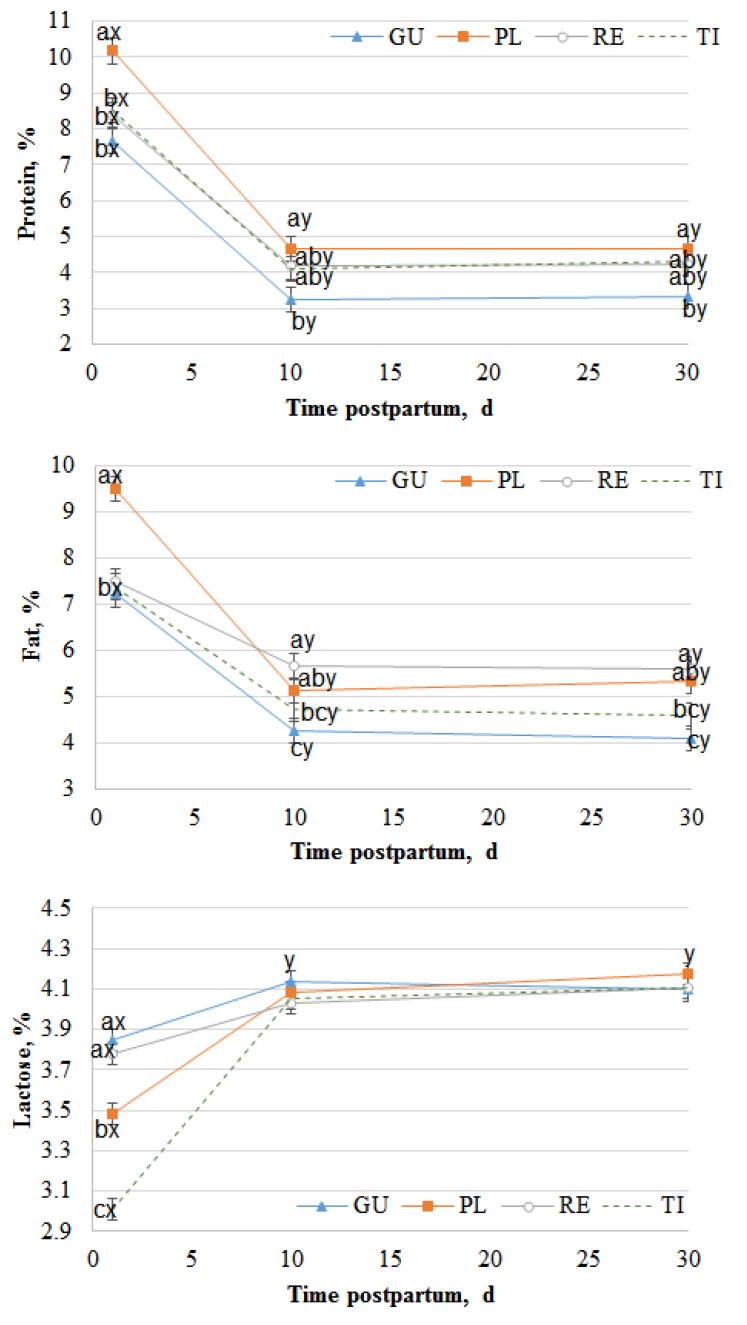
Chemical composition of natural milk at 1, 10 and 30 days of lactation. GU, Cabra del Guadarrama; PL, Palmera; RE, Retinta; TI, Tinerfeña. Different superscripts (a, b, c) indicate significant differences within a time of lactation (*P* < 0.05). Different superscripts (x,y) indicate significant differences within a breed (*P* < 0.05).

The chemical composition of natural milk through the first 30 days of lactation is shown in Figure 1. The milk protein percentage was significantly affected by breed and time of lactation (*P* < 0.0001). However, the milk fat and lactose percentages were affected by the interaction between breed and time of lactation (*P* < 0.0001). 

The protein content was higher on the first day of lactation (colostrum) than at the other studied times, especially for the Palmera breed, which presented higher values than the other breeds (*P* < 0.05). Thereafter, the protein percentage decreased significantly (*P* < 0.05) for all the breeds from the 1st to the 10th day of lactation and remained steady from the 10th to the 30th day (*P* > 0.05). Similarly, the fat percentage decreased from the 1st day to the 10th day of lactation, with Palmera presenting a more acute decrease from the 1st day to the 10th day than the other breeds. Conversely, for protein and fat, the lactose percentage increased significantly from the 1st day to the 10th day (*P* < 0.05) and remained constant thereafter, without differences among breeds (*P* > 0.05). The fatty acid composition is shown as g/100 g of FAMES in Table 1, Table 2, Table 3 and Table 4. There were 17 straight SFAs in the milk (Table 1), with C14:0, C16:0 and C18:0 being the most abundant, followed by C10:0 and C12:0. The less abundant fatty acids were mainly odd fatty acids such as C7:0, C9:0, C13:0, C22:0 and C23:0. All SFAs were affected by the interaction of breed and time of lactation (*P* < 0.01), except C17:0.

Independent of the breed, the percentages of C6:0, C8:0, C10:0, C11:0, C12:0 and C13:0 increased from the 1st day to the 30th day of lactation. In the same way, the percentages of C17:0 decreased over time independent of the breed. Nevertheless, the percentages of C4:0, C7:0 and C9:0 increased with lactation time in all breeds except RE, which remained constant, and C15:0 increased in all breeds except GU, which remained constant. In contrast, C14:0 percentages increased only in TI, C16:0 percentages decreased only in GU, and C23:0 percentages increased only in RE. Finally, C20:0 and C22:0 increased over time in GU and decreased in TI without changes in PL and RE, and C18:0 increased in GU, decreased in PL and remained constant in RE and TI. The 17 detected MUFAs are shown in Table 2. C18:1 c9 had the highest percentage and was affected by the magnitude of the interaction between breed and time of lactation (*P* = 0.001). The percentage of C18:1 c9 decreased for all breeds, but the decrease in PL and TI was twofold compared to the decrease in GU and RE. C18:1t11 remained constant with the time of lactation in RE and TI but increased in GU and decreased in PL. The following most abundant fatty acids were the isomers of C16:1. Those isomers, in general, decreased with the time of lactation, although the C16:1c7 of GU and RE remained constant (*P* > 0.05). The time of lactation did not influence the percentages of C18:1t15, C18:1c11 and C18:1c12 of PL and TI, but the percentages of these fatty acids of RE increased with the time of lactation (*P* < 0.05).

Most of the 16 detected PUFAs (Table 3) were significantly affected by the interaction between breed and time of lactation. The most predominant PUFA was C18:2 n–6, which decreased from the 1st day to the 30th day of lactation for all breeds, although PL registered the most pronounced decrease. The FAs showed the following order in terms of their quantities: C18:2 c9, t11, C18:3 n–3 and C20:4 n–6. These FAs also decreased from the 1st day to the 30th day of lactation for all breeds but in different amounts. FA C22:5 n–3 was also relatively abundant in milk at one day of lactation, but its amount was dramatically reduced at 30 days.

Table 4 shows the main groups of straight and branched fatty acids of milk at the 1st day and the 30th day of lactation. All the studied groups were affected by the interaction between breed and time of lactation (*P* < 0.005). Independent of the breed, the SFA content increased as lactation time increased (*P* > 0.05). At 30 days, PL and TI had higher SFA contents than GU and RE (*P* < 0.05). RE and TI did not show changes in the contents of ΣBCFA with time (*P* > 0.05), but GU showed increases, while PL showed decreases with time (*P* < 0.05). The content of Σ*iso*-BCFA was affected by lactation time only in GU, showing decreases over time. On the first day of lactation, there were no differences between breeds for the contents of Σ*anteiso*-BCFA, whereas in PL and RE, it increased from the 1st day to the 30th day (*P* < 0.05), and no changes over time were observed in GU and TI. For all breeds, MUFA and Σ*cis*-MUFA decreased with the time of lactation (*P* < 0.05), with the decrease being more intense for PL and TI than GU and RE. Nevertheless, the Σtrans-MUFA content was unaffected by time in GU, RE and TI (*P* > 0.05), but it decreased in PL. The ΣCLA content changed, showing decreases, with time only in Pl (*P* < 0.05). For all the breeds, the total PUFA content decreased with the time of lactation (*P* < 0.05), but this decrease was smaller in RE than in the other breeds. The n–6:n–3 ratio decreased with time of lactation (*P* < 0.05), except in RE, in which no changes were observed (*P* < 0.05).

**Figure 2:**
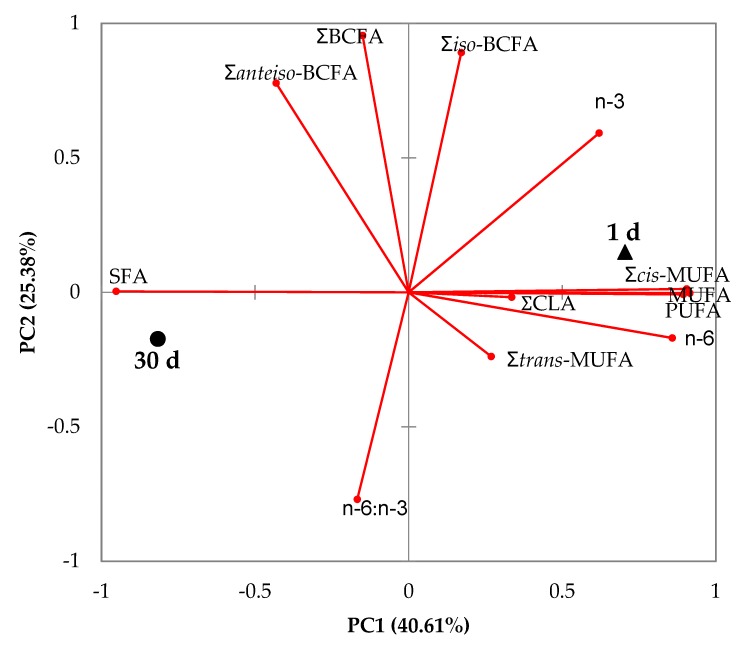
Principal component analysis of the main groups and branched fatty acids of goat milk at 1 and 30 days of lactation. SFA, saturated fatty acids; BCFA, branched chain fatty acids; MUFA, monounsaturated fatty acids; CLA, conjugated linoleic acid; PUFA, polyunsaturated fatty acids.

The biplot of the principal component analysis is shown in Figure 2. The first two principal components summarized approximately 66% of the variation in the data. Milks at the 1st day and the 30th day were clearly discriminated by the variables used in the first principal component. Therefore, milk on the first day was positively related to Σ*cis*-MUFA, ΣMUFA and ΣPUFA, while milk at 30 days was correlated with ΣSFA.

### 3.2. Meat

The 15 detected straight SFAs are shown in Table 5. The predominant SFAs were C14:0 and C16:0, and C18:0. C9:0, C11:0, C12:0, C14:0, C16:0, C18:0 and C22:0 were affected by the interaction between breed and rearing system (*P* < 0.01), whereas C6:0 and C8:0 were not affected by any of the study effects (*P* > 0.05). In general terms, meat from suckling kids fed natural milk had higher values of C15:0, C16:0, C17:0 and C18:0 than meat from kids fed milk replacers (*P* < 0.05), and the effect of rearing system was more noticeable for TI, in which 9 of the 15 acids were affected, than in the other breeds. The most abundant MUFA (Table 6) was C18:1c9, which was affected by the interaction between breed and rearing system (*P* < 0.001). GU fed natural milk had higher contents and RE lower contents of this FA than kids fed milk replacers (*P* < 0.05), whereas the rearing system did not affect PL and TI (*P* > 0.05). There were 16 detected PUFAs, as shown in Table 7. The most abundant PUFA was C18:2 n–6, which was affected by the interaction between rearing system and breed (*P* < 0.01). Meat from PL, RE and TI kids fed natural milk had a lower content of C18:2 n–6 than meat from kids fed milk replacers, without differences between rearing systems (*P* > 0.05). The following fatty acid in quantity was C20:4 n–6. This fatty acid was only affected by breed (*P* = 0.0001), having higher values in GU than the rest of the breeds, especially when reared with milk replacers.

The main groups of straight and branched fatty acids are shown in Table 8. SFAs of meat from GU, PL and TI were similar in both rearing systems (*P* > 0.05), but RE had a higher ΣSFA content when fed natural milk (*P* < 0.05). The *iso*-, *anteiso*- and total BCFAs were affected by the interaction of the principal effects (*P* < 0.01). Hence, for PL and RE, the total BCFA amount was higher when animals were fed natural milk, whereas no effect of the rearing system was observed for GU and TI. All the breeds presented higher *iso*-BCFA amounts when fed natural milk except TI, for which no rearing system effects were observed, and regarding *anteiso*, only RE was affected by the rearing system, with higher amounts when animals were fed natural milk. (*P* < 0.05). The rearing system did not affect the ΣMUFAs of GU, PL and TI (*P* > 0.05). However, the meat of RE had lower ΣMUFAs when kids were fed natural milk (*P* < 0.05). The ΣCLA was higher in PL and RE when kids were fed natural milk (*P* < 0.05) but remained constant in meat of GU and TI (*P* < 0.05). PUFAs were affected by breed and rearing system (*P* < 0.001). Therefore, in general terms, meat from suckling kids fed natural milk had lower amounts of PUFAs. Finally, the desirable fatty acids were affected by the interaction between rearing system and breed (*P* < 0.001). Therefore, RE had the most desirable fatty acids when fed milk replacers but this index diminished when they were fed natural milk. Conversely, GU had the highest desirable fatty acid index when fed natural milk. PL and TI were not influenced by the rearing system (*P* > 0.05).

**Figure 3:**
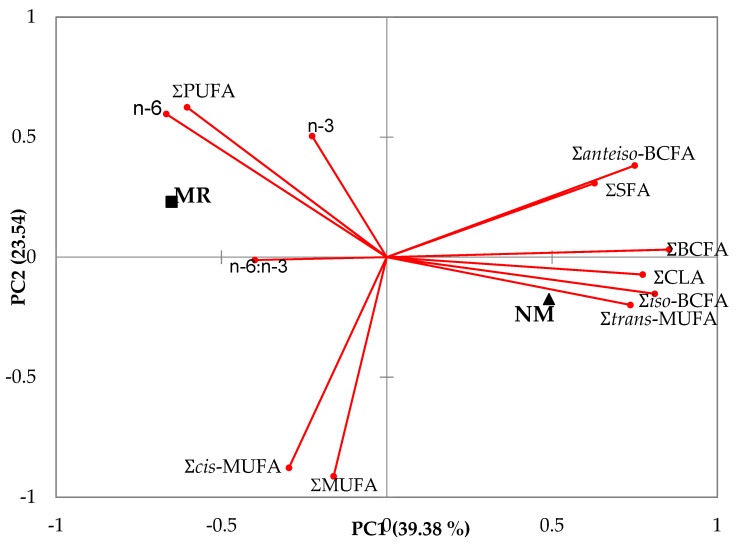
Principal component analysis of the main groups and branched fatty acids of meat of kids reared with milk replacers (MR) or natural milk (NM). SFA, saturated fatty acids; BCFA, branched chain fatty acids; MUFA, monounsaturated fatty acids; CLA, conjugated linoleic acid; PUFA, polyunsaturated fatty acids.

Figure 3 shows the principal component analysis of the main groups and branched fatty acids as a function of the rearing system. The two first principal components summarized almost 63% of the variation in the data (Figure 3). Feeding suckling kids NM was positively related to ΣBCFA, ΣCLA, Σ*iso*-BCFA and Σtrans-MUFA, and negatively related to n–6:n–3 in their meat. However, feeding suckling kids MR was related to n–6 and PUFAs in their meat.

## 4. Discussion

### 4.1. Milk

It is well reported that milk composition changes in the first few days of lactation and that goat colostrum has more protein and fat and less lactose than milk [26,27,28,29]. In agreement with this, in the current experiment, protein and fat percentages decreased and the lactose percentage increased from partum to 10 d of lactation. However, once colostrum production ceased, the milk composition was almost constant over time because the mammary gland developed a high tolerance to external factors, mainly diet [30,31], over the course of evolution to preserve functions and ensure the survival of the newborn ruminants [32]. In addition, this resilience is extendable to the fatty acid composition [33]. Current results for the proximate composition of colostrum are in accordance with those reported in the Majorera breed, with values of protein ranging from 7%–10%, fat ranging from 8%–9% and lactose ranging from 2%–4% [34,35]. Similar results for protein, fat and lactose (6.2%, 7.4% and 4.1%, respectively) were also reported for Murciano-Granadina goats [36]. However, low values of protein and fat were described in Tinerfeña colostrum [37]. Regarding the milk composition, we found higher protein and fat percentages but lower lactose percentages than the milk of Majorera and Payoya at comparable times of lactation reported by other authors [26,27,38]. However, the milk of Sarda had similar percentages of protein and fat but higher lactose content than that of other breeds [39]. Because goat milk has lower percentages of lactose than cow milk, it can be an alternative to people with lactose-related health problems.

Literature comparing fatty acids of goat colostrum and milk is scarce. Lou et al. [40] reported higher values of SFAs and lower values of MUFAs in milk than in colostrum of Laoshan breeds, which agreed with our results. Additionally, these latter authors [39] reported that the SFA proportion in colostrum ranged from 70% to 80%, in agreement with our results, while Marziali et al. [41] reported that the SFA proportion in colostrum of Murciano-Granadina ranged from 48% to 58%. On the other hand, it can be seen in Table 4 that this decrease in the total MUFA amount was mainly due to a decrease in the cis-MUFA series, whereas the change was less noticeable for trans-MUFAs. Similarly, there was a decrease in the total PUFA amount, whereas the n6 amount was lower in milk than in colostrum for all the studied breeds. However, the n3 amount only decreased from colostrum to milk in GU and TI. Therefore, both the relative proportions of different groups of fatty acids as well as the kind of fatty acids differ between colostrum and milk, which could be reflected in the kid meat composition. Ruminant milk is the greatest source of CLA [30], and C18:1c9 is one of the most important fatty acids detected both in milk and colostrum, as reported by Marziali, Guerra, Cerdán-Garcia, Segura-Carretero, Caboni and Verardo [41]. As reported by previous researchers [42,43], cis- and trans- C18:1 acids are often detected in goat milk, with a preeminent occurrence of C18:1 t11. However, from a sensory point of view, C8:0, C10:0 and 4-methyloctanoic fatty acids were the most influential in the flavor of milk and more predominant in caprine than in bovine or ovine milk [40,44]. Monomethyl-branched substitutions on short-chain fatty acids (C4-C6) are found only in goat milk and are implicated in goat-like flavors [45]. Regarding branched fatty acids, LeDoux, Rouzeau, Bas and Sauvant [42] also reported values of approximately 2.2% in goat milk, whereas Massart-Leën et al. [46] found that branched chain fatty acids comprised 2% of goat milk and 3% of cow milk. These values are much higher than those found in the present study. BCFAs in milk fat are derived from the incorporation of BCFAs of rumen bacterial lipids, whereas endogenous synthesis is limited [47]. The discrepancies in the results of this study and previous results could be derived from several factors, such as the diets used, and deserves more attention in the future. In literature, several fatty acids have been widely studied for several reasons. For example, linoleic and alpha linolenic acid are the only essential fatty acids [48], and some long-chain fatty acids (docosahexaenoic, eicosapentaenoic and arachidonic) are precursors of bioactive molecules such as prostaglandins, thromboxanes, leukotrienes and others [49]. In addition, as pointed out by Vlaeminck, Fievez, Cabrita, Fonseca and Dewhurst [47], CLA is generally a lesser component of milk fat than BCFA, but both have similar potential activity against cancer. Although milk and dairy products have a high SFA content, it was concluded that the effects on human cardiovascular health are neutral or even positive [50]. It is possible that BCFAs play an important role in health benefits and must be investigated deeply.

### 4.2. Meat

The rumen of goat kids is fully functional only at approximately 56 days of age [51]. Therefore, ruminal biohydrogenation is limited or nonexistent until this time [52], so the fatty acid content of the intramuscular fat of kids should be influenced largely by dietary fatty acids (milk). Because 6%–20% of the de novo fatty acids synthetized arise mainly in the mammary gland and adipose tissue, the fatty acids of intramuscular fat should be influenced largely by the diets of the dams [52,53]. The endogenous synthesis of MUFA is specifically catalyzed by the ∆-9 desaturase enzyme. It has been reported that mammary desaturase activity is higher in dairy animals than in meat animals [54,55,56]. However, in the present study, PL and TI, which are both dairy breeds, had higher percentages of *trans* MUFAs and most of the C18:1 isomers but lower amounts of the total MUFAs than RE (meat) and GU (mixed purpose). It was reported that meat kids show a higher percentage of CLA than dairy kids [57]. In the current results, the most abundant C18:1 isomers were C18:1c9 and C18:1 t11, in agreement with Adeyemi et al. [58]. These fatty acids are important because C18:1c9 decreases the blood cholesterol content [59], while an anti-atherogenic effect of 18:1 c9t11 has been presumed [60].

It is well known that the n–6:n–3 ratio is an indicator of the role of fatty acids in coronary heart diseases. Although the effects of the ratio on human health are less consistent than expected [61], it is accepted that this ratio must be lower than four [62]. Current results for kid meat varied between 4.78 and 13.00, in agreement with those of other authors [57]. The intramuscular fat from dairy animals, such as PL and TI, had a more unfavorable n–6:n–3 ratio than other breeds [55]. Although some authors did not find a relationship between slightly different milks and intramuscular fatty acids [63], suckling kid goats have a less healthy n–6:n–3 ratio than adult goats fed forages [58].

Several authors reported that fatty acid profiles in the fat of suckling kids usually reflect the fatty acid profiles of the suckled meat [64,65]. Although the lack of the chemical and fatty acid composition of milk replacers is a potential limitation of this study, we want to draw attention to the fact that commercial milk replacers for kids used were very similar between breeds. In addition, the chemical composition of the milk replacer is the same for all kids while the composition of the fat of the kids varies. Tsiplakou et al. [66] reported that the FA profile of goat kids’ muscles reflected that of suckled milk (natural or artificial) and the FA of muscle might help us to discover when artificially reared goat kids are sold as naturally suckled kids. According to De Palo et al. [67], artificial feeding could increase the amount of unsaturated fatty acids to improve its profitability of milk. Other authors reported that the muscle FA profile of goat kids is healthier when fed natural milk that when fed milk replacers [66], although this is related with the composition of milk replacer. Hence, the modification of the fatty acid composition of milk replacers can be a useful tool to improve the quality of the intermuscular fat of suckling kids. Supplementation of milk replacers with docohexanoic acid increased the DHA concentration and the n–6/n–3 ratios were reduced in tissues of goats kids. However, some fatty acids of muscle are less prone to be modified than others. Joy et al. [68] reported similar conclusions in suckling lambs. Therefore, CLA and n–6/n–3 of natural milk and muscles had high correlations while SFA and PUFA n–3 had low correlations. Other authors found that same positive relationship between linolenic acid in milk and meat and the lack of relationship between C18:2 n–6 in milk and meat [69]. Other authors found that same positive relationship between linolenic acid in milk and meat and the lack of relationship between C18:2 n–6 in milk and meat [69]. However, to modify the fatty acids of the suckled natural milk is mandatory to modify the goats’ diets. Hence, the feeding affected to the PUFA, CLA c9,t11, PUFA/SFA, PUFA n–3 and PUFA n–6/n–3 of sheep milk, revealing higher CLA, PUFA/SFA, PUFA n–3 and PUFA n–6/n–3 in milk and suckling lamb meat when grazing pastures instead of being hay fed. Sanz-Sampelayo et al. [70] investigated the possibility of improving the composition of goat meat, in terms of the fatty acid composition, using concentrates supplemented with polyunsaturated fatty acids. These authors reported that milk contained fat with a lower content of saturated fatty acids and a higher content of n–3 PUFA, trans-C18: 1 and CLA. The intramuscular fat presented of the suckling kids had a higher proportion of n–3 PUFA, trans C18: 1 and CLA, while that of n–6 PUFA remained unchanged. In consequence, it is demonstrated that important fatty acids of milk can be improved by modifying the goats’ diets and the modified milk can improve the fatty acid composition of the intramuscular fat of the suckling kids

## 5. Conclusions

This study confirmed that a great change in composition occurs from colostrum to milk. Once colostrum production has finished, milk composition is almost constant over time, demonstrating that the mammary gland has developed a high tolerance to external factors, such as diet. Goat colostrum has more protein and fat and less lactose than milk. The amount of lactose is actually lower in goat milk than in cow milk, which is of interest to people with lactose tolerance issues. Goat milk is an important source of healthy fatty acids such as C18:1 c9 and C18:2 n–6. The percentage of C18:1 c9 decreased from colostrum to milk for all breeds, but the decrease in PL and TI (dairy breeds) was twofold compared to the decrease in GU and RE (meat breeds).

Suckling kid meat was also an important source of C18:1c9. Goat dairy breeds had higher percentages of *trans* MUFAs and most of the C18:1 isomers but lower amounts of total MUFAs. However, these dairy kids had meat with a lower percentage of CLA than meat kids. The meat of kids fed natural milk had higher amounts of CLA and BCFA and lower amounts of n–6 fatty acids than kids fed milk replacers. The presence of BCFAs on the meat of preruminant animals could be explained by the intake of BCFAs. Both milk and meat are a source of linoleic and alpha linolenic acids, which are essential fatty acids and healthy long-chain fatty acids, such as DHA, EPA and ARA. The effect of the rearing system on the fatty acid composition of milk and meat is clearly modulated by the breed. Therefore, investigations related to this topic should be afforded using more than one breed to avoid results open to misinterpretation. 

Regarding the possibility of improving the fatty acid composition of suckling kid fat, several authors reported that the fatty acid profile in the fat of suckling kids usually reflects the fatty acid profile of the suckled meat [63,64].

Additionally, a lack of knowledge on the presence of branched chain fatty acids in both milk and meat of suckling kids was identified. Due to the rising importance of these fatty acids on human health, and the contribution to their flavor, more attention is warranted in future research.

## Figures and Tables

**Table 1:** Individual straight saturated fatty acids of goat milk at 1 and 30 days of lactation (g/100 g of fatty acid methyl esthers (FAMEs)).

Time (T)	1 d of Lactation				30 d of Lactation							
Breed (B)	GU ^†^	PL	RE	TI	GU	PL	RE	TI	s.e.	B	T	B × T
C4:0	1.48^c^	1.06^d^	1.72^b^	1.27^cd^	2.03^a^	1.62^bc^	1.77^ab^	2.02^a^	0.103	0.005	0.0001	0.002
C6:0	1.44^d^	1.01^e^	1.79^c^	1.21^de^	2.37^ab^	2.22^ab^	2.20^b^	2.54^a^	0.110	0.042	0.0001	0.0001
C7:0	0.012^bc^	0.010^bc^	0.012^bc^	0.005^c^	0.051^a^	0.048^a^	0.023^b^	0.062^a^	0.006	0.018	0.0001	0.001
C8:0	1.57^c^	1.08^d^	2.07^b^	1.18^d^	2.85^a^	2.94^a^	2.78^a^	2.86^a^	0.135	0.005	0.0001	0.0001
C9:0	0.035^cd^	0.031^cd^	0.040^cd^	0.025^d^	0.112^b^	0.130^ab^	0.068^c^	0.146^a^	0.012	0.031	0.0001	0.0001
C10:0	4.76^d^	3.42^d^	6.35^c^	3.51^d^	8.75^b^	11.18^a^	9.53^ab^	10.13^a^	0.483	0.020	0.0001	0.0001
C11:0	0.125^e^	0.091^e^	0.176^d^	0.080^e^	0.273^bc^	0.376^a^	0.252^c^	0.314^ab^	0.020	0.40	0.0001	0.0001
C12:0	2.48^de^	1.85^ef^	2.92^d^	1.73^f^	3.52^c^	4.98^a^	4.18^ab^	4.00^bc^	0.241	0.007	0.0001	0.0001
C13:0	0.087^c^	0.059^cd^	0.077^c^	0.041^d^	0.153^ab^	0.193^a^	0.131^b^	0.157^ab^	0.013	0.12	0.0001	0.007
C14:0	11.29^ab^	9.90^ab^	11.84^a^	9.50^b^	10.06^ab^	12.52^a^	11.76^a^	12.07^a^	0.694	0.23	0.059	0.007
C15:0	0.593^b^	0.530^b^	0.618^b^	0.521^b^	0.635^b^	0.945^a^	0.818^a^	0.954^a^	0.049	0.012	0.0001	0.0001
C16:0	24.37^b^	27.88^a^	24.47^b^	26.86^a^	19.62^c^	27.83^a^	23.85^b^	28.24^a^	0.718	0.0001	0.057	0.0001
C17:0	0.804^bcd^	0.955^ab^	0.879^abc^	0.965^a^	0.540^e^	0.746^cd^	0.708^d^	0.698^d^	0.047	0.0001	0.0001	0.60
C18:0	13.30^b^	12.31^bcd^	10.28^de^	12.91^bc^	17.64^a^	9.25^e^	12.29^bcd^	10.87^cde^	0.663	0.0001	0.52	0.0001
C20:0	0.212^b^	0.190^bcd^	0.171^cd^	0.221^b^	0.338^a^	0.150^cd^	0.191^bc^	0.140^d^	0.015	0.0001	0.56	0.0001
C22:0	0.050^bc^	0.047^bcd^	0.048^bcd^	0.057^bc^	0.074^a^	0.037^cd^	0.060^b^	0.034^d^	0.005	0.001	0.83	0.0001
C23:0	0.006^c^	0.008^c^	0.015^b^	0.017^b^	0.006^c^	0.008^c^	0.022^a^	0.014^b^	0.002	0.0001	0.47	0.002

^†^ GU, del Guadarrama; PL, Palmera; RE, Retinta; TI, Tinerfeña; s.e., standard error. Different superscripts in the same row indicate significant differences (*P* ≤ 0.05).

**Table 2:** Individual monounsaturated fatty acids of goat milk at 1 and 30 days of lactation (g/100 g of FAMEs).

Time (T)	1 d of Lactation				30 d of Lactation							
Breed (B)	GU ^†^	PL	RE	TI	GU	PL	RE	TI	s.e.	B	T	B × T
C12:1	0.050	0.032	0.034	0.023	0.037	0.050	0.047	0.035	0.015	0.47	0.31	0.38
C14:1c9	0.078^bc^	0.066^bcd^	0.103^a^	0.062^cd^	0.055^d^	0.084^abc^	0.084^ab^	0.063^cd^	0.007	0.0001	0.242	0.025
C15:1	0.011	0.004	0.005	0.005	0.012	0.005	0.005	0.005^a^	0.005	0.33	0.942	0.99
C16:1t9	0.361^cd^	0.445^ab^	0.487^a^	0.444^ab^	0.272^e^	0.377^cd^	0.404^bc^	0.336^d^	0.049	0.0001	0.0001	0.79
C16:1c7	0.338^abc^	0.358^ab^	0.344^abc^	0.385^a^	0.293^bcd^	0.264^d^	0.274^cd^	0.237^d^	0.022	0.99	0.0001	0.061
C16:1c9	1.185^b^	1.487^a^	1.495^a^	1.588^a^	0.727^d^	1.070^bc^	1.056^bc^	0.965^c^	0.063	0.0001	0.0001	0.323
C17:1c9	0.337^b^	0.353^b^	0.537^a^	0.512^a^	0.142^d^	0.205^cd^	0.290^bc^	0.252^bcd^	0.037	0.0001	0.0001	0.50
C18:1t11	0.839^c^	1.257^a^	0.683^c^	0.717^c^	1.157^ab^	0.674^c^	0.817^c^	0.901^bc^	0.104	0.024	0.86	0.004
C18:1c9	27.217^b^	29.100^ab^	27.559^ab^	30.274^a^	22.953^c^	17.745^de^	21.396^cd^	17.713^e^	1.186	0.58	0.0001	0.0001
C18:1t15	0.125^bc^	0.120^bc^	0.128^bc^	0.121^bc^	0.156^ab^	0.089^c^	0.171^a^	0.116^c^	0.011	0.000	0.22	0.007
C18:1c11	0.173^b^	0.123^bc^	0.117^c^	0.114^c^	0.239^a^	0.142^bc^	0.163^b^	0.136^bc^	0.015	0.0001	0.001	0.32
C18:1c12	0.139^ab^	0.139^ab^	0.074^c^	0.082^c^	0.159^a^	0.105^bc^	0.121^b^	0.079^c^	0.010	0.0001	0.33	0.005
C18:1c13	0.047	0.055	0.037	0.041	0.047	0.046	0.057	0.039	0.006	0.41	0.631	0.11
C18:1t16	0.146^ab^	0.139^ab^	0.134^b^	0.130^b^	0.153^ab^	0.116^bc^	0.168^a^	0.100^c^	0.011	0.001	0.69	0.010
C18:1c15	0.066^ab^	0.058^ab^	0.060^ab^	0.059^ab^	0.059^ab^	0.048^b^	0.070^a^	0.050^b^	0.005	0.038	0.23	0.12
C20:1n–9	0.007	0.002	0.002	0.001	0.001	0.001	0.002	0.001^a^	0.003	0.49	0.30	0.47
C22:1	0.012^d^	0.015^cd^	0.022^ab^	0.026^a^	0.003^e^	0.007^e^	0.019^bc^	0.015^cd^	0.002	0.0001	0.0001	0.065

^†^ GU, del Guadarrama; PL, Palmera; RE, Retinta; TI, Tinerfeña; s.e., standard error. Different superscripts in the same row indicate significant differences (*P* ≤ 0.05).

**Table 3:** Individual polyunsaturated fatty acids of goat milk at 1 and 30 days of lactation (g/100 g of FAMEs).

Time (T)	1 d of Lactation				30 d of Lactation							
Breed (B)	GU ^†^	PL	RE	TI	GU	PL	RE	TI	s.e.	B	T	B × T
C18:2 n–6 t9,12	0.122^abc^	0.111^bcd^	0.107^bcd^	0.097^cd^	0.130^ab^	0.087^d^	0.149^a^	0.083^d^	0.009	0.0001	0.67	0.002
C18:2 n–6	3.37^a^	3.31^a^	2.13^c^	2.77^b^	2.62^b^	1.88^cd^	1.78^d^	2.00^cd^	0.120	0.0001	0.0001	0.003
C18:2 c9, t11	0.221^cd^	0.464^a^	0.307^b^	0.254^bc^	0.176^d^	0.248^bcd^	0.293^bc^	0.236^bcd^	0.027	0.0001	0.0001	0.015
C18:2 t9, c11	0.042^b^	0.052^ab^	0.051^ab^	0.047^b^	0.041^b^	0.048^b^	0.059^a^	0.045^b^	0.003	0.0001	0.94	0.20
C18:2 t10, c12	0.019^ab^	0.022^ab^	0.019^ab^	0.019^ab^	0.017^ab^	0.013^b^	0.023^a^	0.017^ab^	0.002	0.35	0.17	0.065
C18:3 n–6	0.031^a^	0.020^ab^	0.012^b^	0.021^ab^	0.009^b^	0.012^ab^	0.005^b^	0.012^b^	0.005	0.11	0.005	0.30
C18:3 n–3	0.418^a^	0.245^bcd^	0.350^ab^	0.265^bc^	0.383^a^	0.223^cd^	0.420^a^	0.171^d^	0.031	0.0001	0.365	0.030
C20:2 n–6	0.027^b^	0.041^a^	0.018^c^	0.029^b^	0.010^d^	0.013^cd^	0.012^d^	0.011^d^	0.002	0.0001	0.0001	0.0001
C20:3 n–9	0.032^b^	0.036^ab^	0.026^cd^	0.042^a^	0.014^f^	0.018^ef^	0.021^de^	0.028^bc^	0.002	0.0001	0.0001	0.016
C20:3 n–6	0.001^c^	0.002^c^	0.006^b^	0.009^a^	0.001^c^	0.003^c^	0.008^ab^	0.008^ab^	0.001	0.0001	0.63	0.43
C20:4 n–6	0.521^a^	0.483^a^	0.356^b^	0.526^a^	0.164^c^	0.190^c^	0.170^c^	0.215^c^	0.029	0.001	0.0001	0.008
C20:5 n–3	0.096^a^	0.071^b^	0.075^b^	0.078^b^	0.048^c^	0.041^c^	0.066^b^	0.036^c^	0.006	0.006	0.0001	0.001
C22:3 n–3	0.013^b^	0.018^ab^	0.015^b^	0.020^a^	0.004^d^	0.012^bc^	0.022^a^	0.006^cd^	0.002	0.0001	0.0001	0.0001
C22:4 n–6	0.084^a^	0.109^a^	0.045^b^	0.089^a^	0.028^bc^	0.026^bc^	0.018^c^	0.025^c^	0.008	0.0001	0.0001	0.007
C22:5 n–3	0.314^a^	0.158^c^	0.201^bc^	0.227^b^	0.074^d^	0.051^d^	0.088^d^	0.053^d^	0.017	0.0001	0.0001	0.0001
C22:6 n–3	0.054^a^	0.016^c^	0.033^b^	0.015^c^	0.014^c^	0.005^c^	0.018^c^	0.006^c^	0.004	0.0001	0.0001	0.0001

^†^ GU, del Guadarrama; PL, Palmera; RE, Retinta; TI, Tinerfeña; s.e., standard error. Different superscripts in the same row indicate significant differences (*P* ≤ 0.05).

**Table 4:** Main groups of straight and branched fatty acids of goat milk at 1 and 30 days of lactation (g/100 g of FAMEs).

Time (T)	1 d of Lactation				30 d of Lactation							
Breed (B)	GU ^†^	PL	RE	TI	GU	PL	RE	TI	s.e.	B	T	B × T
SFA	63.48^c^	61.03^c^	64.37^c^	60.85^c^	69.79^b^	76.07^a^	71.64^b^	75.96^a^	1.384	0.40	0.0001	0.0001
ƩBCFA	0.678^b^	0.475^c^	0.730^ab^	0.564^c^	0.569^c^	0.696^ab^	0.814^a^	0.575^c^	0.035	0.0001	0.046	0.0001
Ʃiso-BCFA	0.404^a^	0.238^c^	0.423^a^	0.320^b^	0.311^b^	0.305^bc^	0.407^a^	0.279^bc^	0.018	0.0001	0.120	0.002
Ʃanteiso-BCFA	0.274^b^	0.237^b^	0.307^b^	0.244^b^	0.258^b^	0.391^a^	0.407^a^	0.296^b^	0.021	0.0001	0.0001	0.001
MUFA	31.13^b^	33.75^ab^	31.82^ab^	34.58^a^	26.47^c^	21.03^d^	25.14^cd^	21.04^d^	1.292	0.72	0.0001	0.0001
Ʃcis-MUFA	29.66^b^	31.79^ab^	30.39^ab^	33.17^a^	24.73^c^	19.77^de^	23.58^cd^	19.59^e^	1.269	0.74	0.0001	0.001
Ʃtrans-MUFA	1.47^bc^	1.96^a^	1.43^bc^	1.41^bc^	1.74^ab^	1.26^c^	1.56^abc^	1.45^bc^	0.122	0.34	0.45	0.009
ƩCLA	0.283^de^	0.538^a^	0.377^b^	0.320^bcd^	0.235^e^	0.309^bcde^	0.376^bc^	0.298^cde^	0.030	0.0001	0.001	0.013
PUFA	5.36^a^	5.16^a^	3.75^c^	4.51^b^	3.73^c^	2.86^d^	3.15^d^	2.95^d^	0.175	0.0001	0.0001	0.000
n–6	4.15^a^	4.080^a^	2.68^cd^	3.54^b^	2.96^c^	2.21^de^	2.14^e^	2.35^de^	0.139	0.0001	0.0001	0.001
n–3	0.895^a^	0.508^cd^	0.674^b^	0.605^bc^	0.523^c^	0.331^de^	0.613^bc^	0.273^e^	0.044	0.0001	0.0001	0.0001
n–6:n–3	4.78^c^	8.14^a^	4.08^cd^	5.95^b^	5.80^b^	6.69^b^	3.80^d^	8.95^a^	0.360	0.0001	0.031	0.0001

^†^ GU, del Guadarrama; PL, Palmera; RE, Retinta; TI, Tinerfeña; s.e., standard error. FA. Fatty acids; SFA, saturated fatty acids; BCFA, branched chain fatty acids; MUFA, monounsaturated fatty acids; CLA, conjugated linoleic acid; PUFA, polyunsaturated fatty acids. Different superscripts in the same row indicate significant differences (*P* ≤ 0.05).

**Table 5:** Individual straight saturated fatty acids of suckling kid meat (g/100 g of FAMEs).

RS ^†^	Milk Replacer				Natural Milk							
Breed (B)	GU	PL	RE	TI	GU	PL	RE	TI	s.e.	B	RS	B × RS
C6:0	0.002^a^	0.005^a^	0.002^a^	0.002^a^	0.002^a^	0.003^a^	0.004^a^	0.003^a^	0.001	0.49	0.72	0.17
C8:0	0.004^b^	0.011^a^	0.005^ab^	0.007^ab^	0.006^ab^	0.007^ab^	0.006^ab^	0.009^ab^	0.002	0.401	0.83	0.30
C9:0	0.013^b^	0.077^a^	0.009^b^	0.001^b^	0.030^ab^	0.048^a^	0.025^ab^	0.049^a^	0.012	0.008	0.16	0.008
C10:0	0.081^d^	0.154^cd^	0.142^cd^	0.196^cd^	0.239^c^	0.511^a^	0.274^bc^	0.407^ab^	0.048	0.034	0.0001	0.10
C11:0	0.018^c^	0.066^ab^	0.012^c^	0.011^c^	0.045^abc^	0.073^ab^	0.036^bc^	0.080^a^	0.012	0.07	0.001	0.036
C12:0	3.14^a^	4.25^a^	0.36^c^	3.75^a^	0.79^bc^	1.12^bc^	0.90^bc^	1.56^b^	0.360	0.0001	0.0001	0.0001
C13:0	0.046^de^	0.091^abc^	0.021^e^	0.065^cd^	0.067^bcd^	0.093^ab^	0.054^cd^	0.107^a^	0.010	0.0001	0.003	0.16
C14:0	12.18^a^	10.78^abc^	7.66^d^	11.65^ab^	8.98^cd^	9.26^cd^	9.79^bc^	8.61^cd^	0.641	0.013	0.006	0.0001
C15:0	0.246^c^	0.403^bc^	0.199^c^	0.452^b^	0.425^b^	0.634^a^	0.455^b^	0.715^a^	0.054	0.0001	0.0001	0.78
C16:0	22.60^cd^	25.12^b^	21.12^d^	24.73^b^	21.21^d^	27.49^a^	23.99^bc^	27.64^a^	0.697	0.0001	0.003	0.001
C17:0	0.448^d^	0.562^bcd^	0.530^cd^	0.691^bc^	0.693^bc^	1.021^a^	0.794^b^	0.977^a^	0.062	0.002	0.0001	0.30
C18:0	10.36^c^	9.48^c^	13.15^ab^	9.79^c^	14.07^a^	13.57^a^	13.09^ab^	11.875^b^	0.640	0.017	0.0001	0.003
C20:0	0.062^ab^	0.050^b^	0.066^ab^	0.061^ab^	0.081^a^	0.063^ab^	0.071^ab^	0.067^ab^	0.006	0.34	0.028	0.54
C22:0	0.056^a^	0.018^b^	0.020^b^	0.011^b^	0.010^b^	0.018^b^	0.023^b^	0.014^b^	0.007	0.035	0.07	0.000
C23:0	0.205^a^	0.072^bc^	0.057^c^	0.043^c^	0.173^ab^	0.049^c^	0.098^bc^	0.047^c^	0.023	0.0001	0.90	0.31

^†^ RS, Rearing system; GU, del Guadarrama; PL, Palmera; RE, Retinta; TI, Tinerfeña; s.e., standard error. Different superscripts in the same row indicate significant differences (*P* ≤ 0.05). The least square means were adjusted for a hot carcass weight (HCW) of 5.02 kg.

**Table 6:** Individual monounsaturated fatty acids of suckling kid meat (g/100 g of FAMEs).

RS ^†^	Milk Replacer				Natural Milk							
Breed (B)	GU	PL	RE	TI	GU	PL	RE	TI	s.e.	B	RS	B × RS
C14:1c9	0.229^bc^	0.266^ab^	0.063^g^	0.276^a^	0.107^f^	0.175^de^	0.143^e^	0.200^cd^	0.015	0.0001	0.0001	0.0001
C15:1	0.002^b^	0.013^a^	0.001^b^	0.003^b^	0.003^b^	0.003^b^	0.004^b^	0.004^b^	0.002	0.14	0.36	0.009
C16:1c7	1.92^a^	0.28^b^	0.85^b^	0.34^b^	1.40^ab^	0.31^b^	0.82^b^	0.31^b^	0.317	0.001	0.59	0.71
C16:1c9	2.86^a^	2.37^ab^	2.55^a^	2.64^a^	2.10^b^	2.42^ab^	2.58^a^	2.65^a^	0.132	0.18	0.1	0.001
C17:1c9	0.147^e^	0.324^cde^	0.238^de^	0.425^bc^	0.182^e^	0.537^ab^	0.379^bcd^	0.626^a^	0.054	0.0001	0.001	0.22
C18:1c11	0.079^d^	0.125^bcd^	0.088^cd^	0.127^bc^	0.191^ab^	0.192^a^	0.150^ab^	0.135^b^	0.014	0.12	0.0001	0.0001
C18:1c12	0.042^d^	0.097^cd^	0.069^d^	0.162^b^	0.145^bc^	0.228^a^	0.154^bc^	0.164^b^	0.018	0.005	0.0001	0.0001
C18:1c13	0.010^c^	0.035^b^	0.021^bc^	0.040^b^	0.026^bc^	0.065^a^	0.036^b^	0.035^b^	0.006	0.001	0.004	0.017
C18:1c15	0.026^c^	0.069^ab^	0.016^c^	0.075^ab^	0.032^c^	0.080^a^	0.052^b^	0.053^b^	0.007	0.0001	0.86	0.0001
C18:1t11	0.349^c^	0.680^abc^	0.303^c^	0.929^ab^	0.651^abc^	1.207^a^	0.592^bc^	1.049^ab^	0.169	0.031	0.022	0.63
C18:1t15	0.131^a^	0.137^a^	0.134^a^	0.141^a^	0.173^a^	0.159^a^	0.139^a^	0.141^a^	0.014	0.55	0.12	0.26
C18:1t16	0.018^c^	0.079^bc^	0.035^c^	0.120^b^	0.134^b^	0.216^a^	0.125^b^	0.136^b^	0.021	0.054	0.0001	0.006
C18:1c9	30.51^c^	33.78^b^	41.53^a^	35.65^b^	36.30^b^	33.61^b^	36.477^b^	35.661^b^	1.088	0.0001	0.86	0.0001
C20:1n–9	0.002^a^	0.003^a^	0.002^a^	0.002^a^	0.004^a^	0.001^a^	0.003^a^	0.004^a^	0.001	0.81	0.71	0.32
C22:1	0.010 ^a^	0.003 ^b^	0.010 ^ab^	0.006 ^b^	0.010 ^ab^	0.004 ^b^	0.007 ^ab^	0.004 ^b^	0.002	0.010	0.44	0.71

^†^ RS, Rearing system; GU, del Guadarrama; PL, Palmera; RE, Retinta; TI, Tinerfeña; s.e., standard error. Different superscripts in the same row indicate significant differences (*P* ≤ 0.05).

**Table 7:** Individual polyunsaturated fatty acids of suckling kid meat (g/100 g of FAMEs).

RS ^†^	Milk Replacer				Natural Milk							
Breed (B)	GU	PL	RE	TI	GU	PL	RE	TI	s.e.	B	RS	B × RS
C18:2c9,t11	0.174^cd^	0.264^bcd^	0.097^d^	0.343^b^	0.159^d^	0.500^a^	0.270^bc^	0.353^b^	0.046	0.002	0.006	0.007
C18:2 n–6	7.275^a^	6.048^ab^	7.428^a^	4.465^b^	6.339^a^	2.765^c^	4.283^bc^	3.188^c^	0.468	0.0001	0.0001	0.007
C18:2n–6t9,12	0.024^c^	0.088^b^	0.023^c^	0.118^b^	0.110^b^	0.173^a^	0.108^b^	0.115^b^	0.016	0.010	0.0001	0.001
C18:2t10,c12	0.027^a^	0.026^ab^	0.017^b^	0.025^ab^	0.016^b^	0.021^ab^	0.021^ab^	0.027^a^	0.003	0.177	0.218	0.005
C18:2t9,c11	0.042^a^	0.014^b^	0.023^b^	0.014^b^	0.026^b^	0.011^b^	0.016^b^	0.014^b^	0.004	0.0001	0.077	0.152
C18:3 n–3	0.372^c^	0.230^d^	0.764^a^	0.227^d^	0.435^c^	0.191^d^	0.529^b^	0.193^d^	0.033	0.0001	0.019	0.0001
C18:3 n–6	0.035^a^	0.023^bc^	0.018^bc^	0.025^b^	0.024^bc^	0.020^bc^	0.014^c^	0.023^bc^	0.004	0.000	0.087	0.494
C20:2 n–6	0.125^b^	0.062^c^	0.182^a^	0.020^d^	0.073^c^	0.022^d^	0.059^c^	0.021^d^	0.009	0.0001	0.0001	0.0001
C20:3 n–6	0.003^abc^	0.002^bcd^	0.005^a^	0.001^d^	0.004^ab^	0.001^d^	0.003^abcd^	0.002^cd^	0.001	0.033	0.447	0.303
C20:3 n–9	0.252^a^	0.163^b^	0.093^bcd^	0.074^d^	0.151^bc^	0.078^cd^	0.083^bcd^	0.073^d^	0.022	0.0001	0.005	0.013
C20:4 n–6	3.23^a^	1.95^bc^	0.93^c^	0.87^c^	2.38^b^	1.15^c^	1.25^c^	0.94^c^	0.321	0.0001	0.215	0.097
C20:5 n–3	0.234^a^	0.140^ab^	0.090^b^	0.078^b^	0.169^ab^	0.107^b^	0.179^ab^	0.091^b^	0.029	0.001	0.954	0.020
C22:3 n–3	0.161^a^	0.0001^b^	0.036^b^	0.0001^b^	0.075^b^	0.0001^b^	0.044^b^	0.001^b^	0.025	0.0001	0.386	0.071
C22:4 n–6	0.463^a^	0.264^b^	0.149^b^	0.167^b^	0.227^b^	0.178^b^	0.135^b^	0.171^b^	0.042	0.0001	0.013	0.003
C22:5 n–3	0.598^a^	0.336^bc^	0.235^c^	0.197^c^	0.527^ab^	0.293^c^	0.410^bc^	0.252^c^	0.061	0.0001	0.543	0.117
C22:6 n–3	0.093^a^	0.045^bc^	0.044^c^	0.029^c^	0.109^a^	0.040^c^	0.091^ab^	0.037^c^	0.011	0.0001	0.072	0.127

^†^ RS, Rearing system; GU, del Guadarrama; PL, Palmera; RE, Retinta; TI, Tinerfeña; s.e., standard error. Different superscripts in the same row indicate significant differences (*P* ≤ 0.05). The least square means were adjusted for a hot carcass weight (HCW) of 5.02 kg.

**Table 8:** Main groups of straight and branched fatty acids of suckling kid meat (g/100 g of FAMEs).

RS ^†^	Milk Replacer				Natural Milk							
Breed (B)	GU	PL	RE	TI	GU	PL	RE	TI	s.e.	B	RS	B × RS
ƩSFA	49.87^bc^	51.62^ab^	43.68^d^	51.96^ab^	47.34^c^	54.58^a^	50.21^b^	52.69^ab^	1.216	0.0001	0.047	0.001
ƩBCFA	0.41^c^	0.46^bc^	0.31^d^	0.49^abc^	0.48^abc^	0.60^a^	0.58^ab^	0.51^ab^	0.035	0.30	0.0001	0.001
Ʃiso-BCFA	0.23^cd^	0.27^bc^	0.21^d^	0.31^b^	0.31^b^	0.39^a^	0.39^a^	0.33^ab^	0.024	0.22	0.0001	0.004
Ʃanteiso-BCFA	0.18^a^	0.19^a^	0.11^b^	0.18^a^	0.17^a^	0.21^a^	0.19^a^	0.18^a^	0.015	0.073	0.06	0.001
ƩMUFA	36.48^c^	38.49^bc^	46.03^a^	41.20^b^	41.66^b^	39.65^b^	42.01^b^	41.57^b^	1.126	0.0001	0.45	0.0001
Ʃcis-MUFA	35.84^c^	37.36^bc^	45.43^a^	39.74^b^	40.50^b^	37.63^bc^	40.80^b^	39.85^b^	1.131	0.0001	0.90	0.000
Ʃtrans-MUFA	0.65^c^	1.13^bc^	0.59^c^	1.47^b^	1.16^bc^	2.02^a^	1.21^bc^	1.72^ab^	0.212	0.017	0.001	0.42
ƩCLA	0.24^cd^	0.30^bcd^	0.14^d^	0.38^b^	0.20^cd^	0.53^a^	0.31^bc^	0.39^b^	0.047	0.005	0.014	0.005
ƩPUFA	13.11^a^	9.64^bc^	10.13^b^	6.66^d^	10.83^b^	5.55^d^	7.94^cd^	5.50^d^	0.844	0.0001	0.0001	0.31
n–6	11.16^a^	8.43^bc^	8.73^b^	5.67^d^	9.16^b^	4.31^d^	5.85^cd^	4.46^d^	0.744	0.0001	0.0001	0.18
n–3	1.46^a^	0.74^b^	1.17^a^	0.53^b^	1.32^a^	0.63^b^	1.25^a^	0.57^b^	0.117	0.0001	0.73	0.62
n–6:n–3	7.88^b^	13.00^a^	7.46^b^	12.08^a^	6.88^b^	7.82^b^	4.78^c^	8.88^b^	0.708	0.0001	0.0001	0.02
Desirable FA	59.97^cd^	58.51^cd^	69.24^a^	58.20^d^	65.62^b^	59.28^cd^	61.80^c^	59.28^cd^	0.970	0.0001	0.98	0.0001

^†^ RS, Rearing system; GU, del Guadarrama; PL, Palmera; RE, Retinta; TI, Tinerfeña; s.e., standard error. FA. Fatty acids; SFA, saturated fatty acids; BCFA, branched chain fatty acids; MUFA, monounsaturated fatty acids; CLA, conjugated linoleic acid; PUFA, polyunsaturated fatty acids. Different superscripts in the same row indicate significant differences (*P* ≤ 0.05). The least square means were adjusted for a hot carcass weight (HCW) of 5.02 kg.

## References

[1] FAO (2017). FAOSTAT Livestock Primary.

[2] Yalcintan H., Akin P.D., Ozturk N., Ekiz B., Kocak O., Yilmaz A. (2018). Carcass and meat quality traits of Saanen goat kids reared under natural and artificial systems and slaughtered at different ages. Acta Vet. Brno.

[3] Aparnathi K., Mehta B., Velpula S. (2017). Goat Milk in Human Nutrition and Health—A Review. Int. J. Curr. Microbiol. Appl. Sci..

[4] Castel J.M., Mena Y., Ruiz F.A., Gutiérrez R. (2012). Situación y evolución de los sistemas de producción caprina en España. Tierras Caprino.

[5] Marichal A., Castro N., Capote J., Zamorano N., Arguello A. (2003). Effects of live weight at slaughter (6, 10 and 25 kg) on kid carcass and meat quality. Livest. Prod. Sci..

[6] Argüello A., Castro N., Capote J., Solomon M. (2005). Effects of diet and live weight at slaughter on kid meat quality. Meat Sci..

[7] Ripoll G., Alcalde M.J., Argüello A., Córdoba M.G., Panea B. (2019). Effect of the rearing system on the color of four muscles of suckling kids. Food Sci. Nutr..

[8] Ripoll G., Alcalde M.J., Argüello A., Córdoba M.G., Panea B. (2018). Consumer visual appraisal and shelf life of leg chops from suckling kids raised with natural milk or milk replacer. J. Sci. Food Agric..

[9] Ripoll G., Alcalde M.J., Argüello A., Panea B. (2019). Web-based survey of consumer preferences for the visual appearance of meat from suckling kids. Ital. J. Anim. Sci..

[10] Ripoll G., Córdoba M.G., Alcalde M.J., Martín A., Argüello A., Casquete R., Panea B. (2019). Volatile organic compounds and consumer preference for meat from suckling goat kids raised with natural or replacers milk. Ital. J. Anim. Sci..

[11] Ribeiro R.D.X., Medeiros A.N., Oliveira R.L., de Araújo G.G.L., Queiroga R.d.C.d.E., Ribeiro M.D., Silva T.M., Bezerra L.R., Oliveira R.L. (2018). Palm kernel cake from the biodiesel industry in goat kid diets. Part 2: Physicochemical composition, fatty acid profile and sensory attributes of meat. Small Rumin. Res..

[12] PAHO/WHO (2007). Trans. fats free Americas. Conclusions and Recommendations.

[13] Alonso L., Fontecha F.J., Fraga M.J., Juárez M., Lozada L. (1999). Fatty acid composition of caprine milk: Major, branched-chain, and trans fatty acids. J. Dairy Sci..

[14] Woo A., Lindsay R. (1984). Concentrations of major free fatty acids and flavor development in Italian cheese varieties. J. Dairy Sci..

[15] Watkins P.J., Kearney G., Rose G., Allen D., Ball A.J., Pethick D.W., Warner R.D. (2014). Effect of branched-chain fatty acids, 3-methylindole and 4-methylphenol on consumer sensory scores of grilled lamb meat. Meat Sci..

[16] Brennand C.P., Ha J.K., Lindsay R.C. (1989). Aroma properties and thresholds of some branched-chaiin and other minor volatile fatty acids occurring in milk fat and meat lipids. J. Sens. Stud..

[17] Ran-Ressler R.R., Devapatla S., Lawrence P., Brenna J.T. (2008). Branched chain fatty acids are constituents of the normal healthy newborn gastrointestinal tract. Pediatric Res..

[18] Yang Z., Liu S., Chen X., Chen H., Huang M., Zheng J. (2000). Induction of apoptotic cell death and in vivo growth inhibition of human cancer cells by a saturated branched-chain fatty acid, 13-methyltetradecanoic acid. Cancer Res..

[19] Duncan W.R.H., Garton G.A. (2007). Differences in the proportions of branched-chain fatty acids in subcutaneous triacylglycerols of barley-fed ruminants. Br. J. Nutr..

[20] Serra A., Mele M., La Comba F., Conte G., Buccioni A., Secchiari P. (2009). Conjugated Linoleic Acid (CLA) content of meat from three muscles of Massese suckling lambs slaughtered at different weights. Meat Sci..

[21] E.U. (2010). Directive 2010/63/EU of the European Parliament and of the Council of 22 September 2010 on the protection of animals used for scientific purposes. Off. J. Eur. Union L.

[22] E.U. (2009). Council Regulation (EC) No 1099/2009 of 24 September 2009 on the protection of animals at the time of killing. Off. J. Eur. Union.

[23] Molkentin J., Precht D. (2000). Validation of a gas-chromatographic method for the determination of milk fat contents in mixed fats by butyric acid analysis. Eur. J. Lipid Sci. Technol..

[24] Folch J., Lees M., Stanley G. (1957). A simple method for the isolation and purification of lipids from animal tissues. J. Biol. Chem..

[25] Huerta-Leidenz N., Cross H., Lunt D., Pelton L., Savell J., Smith S. (1991). Growth, carcass traits, and fatty acid profiles of adipose tissues from steers fed whole cottonseed. J. Anim. Sci..

[26] Sanchez-Macias D., Moreno-Indias I., Castro N., Morales-Delanuez A., Arguello A. (2014). From goat colostrum to milk: Physical, chemical, and immune evolution from partum to 90 days postpartum. J. Dairy Sci..

[27] Sanchez-Macias D., Fresno M., Moreno-Indias I., Castro N., Morales-delaNuez A., Alvarez S., Arguello A. (2010). Physicochemical analysis of full-fat, reduced-fat, and low-fat artisan-style goat cheese. J. Dairy Sci..

[28] Linzell J., Peaker M. (1974). Changes in colostrum composition and in the permeability of the mammary epithelium at about the time of parturition in the goat. J. Physiol..

[29] Pecka-Kiełb E., Zachwieja A., Wojtas E., Zawadzki W. (2018). Influence of nutrition on the quality of colostrum and milk of ruminants. Mljekarstvo Časopis za Unaprjeđenje Proizvodnje i Prerade Mlijeka.

[30] Almeida O.C., Ferraz M.V.C., Susin I., Gentil R.S., Polizel D.M., Ferreira E.M., Barroso J.P.R., Pires A.V. (2019). Plasma and milk fatty acid profiles in goats fed diets supplemented with oils from soybean, linseed or fish. Small Rumin. Res..

[31] Bobe G., Zimmerman S., Hammond E.G., Freeman A.E., Porter P.A., Luhman C.M., Beitz D.C. (2007). Butter Composition and Texture from Cows with Different Milk Fatty Acid Compositions Fed Fish Oil or Roasted Soybeans. J. Dairy Sci..

[32] Hernandez-Castellano L.E., Arguello A., Almeida A.M., Castro N., Bendixen E. (2015). Colostrum protein uptake in neonatal lambs examined by descriptive and quantitative liquid chromatography-tandem mass spectrometry. J. Dairy Sci..

[33] Palma M., Alves S.P., Hernandez-Castellano L.E., Capote J., Castro N., Arguello A., Matzapetakis M., Bessa R.J.B., de Almeida A.M. (2017). Mammary gland and milk fatty acid composition of two dairy goat breeds under feed-restriction. J. Dairy Res..

[34] Moreno-Indias I., Sánchez-Macías D., Castro N., Morales-delaNuez A., Hernández-Castellano L.E., Capote J., Argüello A. (2012). Chemical composition and immune status of dairy goat colostrum fractions during the first 10h after partum. Small Rumin. Res..

[35] Argüello A., Castro N., Álvarez S., Capote J. (2006). Effects of the number of lactations and litter size on chemical composition and physical characteristics of goat colostrum. Small Rumin. Res..

[36] Romero T., Beltrán M.C., Rodríguez M., De Olives A.M., Molina M.P. (2013). Short communication: Goat colostrum quality: Litter size and lactation number effects. J. Dairy Res..

[37] Capote J., Castro N., Caja G., Fernández G., Briggs H., Argüello A. (2008). Effects of the frequency of milking and lactation stage on milk fractions and milk composition in Tinerfeña dairy goats. Small Rumin. Res..

[38] Delgado-Pertíñez M., Guzmán-Guerrero J.L., Caravaca F.P., Castel J.M., Ruiz F.A., González-Redondo P., Alcalde M.J. (2009). Effect of artificial vs. natural rearing on milk yield, kid growth and cost in Payoya autochthonous dairy goats. Small Rumin. Res..

[39] Nudda A., Battacone G., Bee G., Boe R., Castanares N., Lovicu M., Pulina G. (2015). Effect of linseed supplementation of the gestation and lactation diets of dairy ewes on the growth performance and the intramuscular fatty acid composition of their lambs. Animal.

[40] Lou X., Li J., Zhang X., Wang J., Wang C. (2018). Variations in fatty acid composition of Laoshan goat milk from partum to 135 days postpartum. Anim. Sci. J..

[41] Marziali S., Guerra E., Cerdán-Garcia C., Segura-Carretero A., Caboni M.F., Verardo V. (2018). Effect of early lactation stage on goat colostrum: Assessment of lipid and oligosaccharide compounds. Int. Dairy J..

[42] LeDoux M., Rouzeau A., Bas P., Sauvant D. (2002). Occurrence of trans-C18: 1 fatty acid isomers in goat milk: Effect of two dietary regimens. J. Dairy Sci..

[43] Andueza D., Rouel J., Chilliard Y., Leroux C., Ferlay A. (2013). Prediction of the goat milk fatty acids by near infrared reflectance spectroscopy. Eur. J. Lipid Sci. Technol..

[44] Ha J.K., Lindsay R. (1993). Release of volatile branched-chain and other fatty acids from ruminant milk fats by various lipases. J. Dairy Sci..

[45] Haenlein G. (2004). Goat milk in human nutrition. Small Rumin. Res..

[46] Massart-Leën A.M., De Pooter H., Decloedt M., Schamp N. (1981). Composition and variability of the branched-chain fatty acid fraction in the milk of goats and cows. Lipids.

[47] Vlaeminck B., Fievez V., Cabrita A.R.J., Fonseca A.J.M., Dewhurst R.J. (2006). Factors affecting odd- and branched-chain fatty acids in milk: A review. Anim. Feed Sci. Technol..

[48] Albenzio M., Santillo A., Avondo M., Nudda A., Chessa S., Pirisi A., Banni S. (2016). Nutritional properties of small ruminant food products and their role on human health. Small Rumin. Res..

[49] Calder P.C. (2011). Fatty acids and inflammation: The cutting edge between food and pharma. Eur. J. Pharmacol..

[50] Lordan R., Tsoupras A., Mitra B., Zabetakis I. (2018). Dairy fats and cardiovascular disease: Do we really need to be concerned?. Foods.

[51] Van Soest P. (1994). Function of the ruminant forestomach. Nutritional Ecology of the Ruminant.

[52] Valvo M.A., Lanza M., Bella M., Fasone V., Scerra M., Biondi L., Priolo A. (2005). Effect of ewe feeding system (grass v. concentrate) on intramuscular fatty acids of lambs raised exclusively on maternal milk. Anim. Sci..

[53] Osorio M.T., Zumalacarregui J.M., Figueira A., Mateo J. (2007). Fatty acid composition in subcutaneous, intermuscular and intramuscular fat deposits of suckling lamb meat: Effect of milk source. Small Rumin. Res..

[54] Wood J.D., Enser M., Fisher A.V., Nute G.R., Sheard P.R., Richardson R.I., Hughes S.I., Whittington F.M. (2008). Fat deposition, fatty acid composition and meat quality: A review. Meat Sci..

[55] Horcada A., Ripoll G., Alcalde M.J., Sanudo C., Teixeira A., Panea B. (2012). Fatty acid profile of three adipose depots in seven Spanish breeds of suckling kids. Meat Sci..

[56] Chilliard Y., Rouel J., Leroux C. (2006). Goat’s alpha-s1 casein genotype influences its milk fatty acid composition and delta-9 desaturation ratios. Anim. Feed Sci. Technol..

[57] Horcada A., Campo M.D.M., Polvillo O., Alcalde M.J., Cilla I., Sañudo C. (2014). A comparative study of fatty acid profiles of fat in commercial Spanish suckling kids and lambs. Span. J. Agric. Res..

[58] Adeyemi K.D., Shittu R.M., Sabow A.B., Karim R., Sazili A.Q. (2017). Myofibrillar protein, lipid and myoglobin oxidation, antioxidant profile, physicochemical and sensory properties of caprine *longissimus thoracis* during *postmortem* conditioning. J. Food Process. Preserv..

[59] Binkoski A.E., Kris-Etherton P.M., Wilson T.A., Mountain M.L., Nicolosi R.J. (2005). Balance of unsaturated fatty acids is important to a cholesterol-lowering diet: Comparison of mid-oleic sunflower oil and olive oil on cardiovascular disease risk factors. J. Am. Diet. Assoc..

[60] Pariza M.W., Park Y., Cook M.E. (2001). The biologically active isomers of conjugated linoleic acid. Prog. Lipid Res..

[61] Marventano S., Kolacz P., Castellano S., Galvano F., Buscemi S., Mistretta A., Grosso G. (2015). A review of recent evidence in human studies of n–3 and n–6 PUFA intake on cardiovascular disease, cancer, and depressive disorders: Does the ratio really matter?. Int. J. Food Sci. Nutr..

[62] Scollan N., Hocquette J.F., Nuernberg K., Dannenberger D., Richardson I., Moloney A. (2006). Innovations in beef production systems that enhance the nutritional and health value of beef lipids and their relationship with meat quality. Meat Sci..

[63] Manso T., Bodas R., Vieira C., Mantecon A.R., Castro T. (2011). Feeding vegetable oils to lactating ewes modifies the fatty acid profile of suckling lambs. Animal.

[64] Bas P., Morand-Fehr P. (2000). Effect of nutritional factors on fatty acid composition of lamb fat deposits. Livest. Prod. Sci..

[65] Bañón S., Vila R., Price A., Ferrandini E., Garrido M.D. (2006). Effects of goat milk or milk replacer diet on meat quality and fat composition of suckling goat kids. Meat Sci..

[66] Tsiplakou E., Papadomichelakis G., Sparaggis D., Sotirakoglou K., Georgiadou M., Zervas G. (2016). The effect of maternal or artificial milk, age and sex on three muscles fatty acid profile of Damascus breed goat kids. Livest. Sci..

[67] De Palo P., Maggiolino A., Centoducati N., Tateo A. (2015). Effects of different milk replacers on carcass traits, meat quality, meat color and fatty acids profile of dairy goat kids. Small Rumin. Res..

[68] Joy M., Ripoll G., Molino F., Dervishi E., Alvarez-Rodriguez J. (2012). Influence of the type of forage supplied to ewes in pre- and post-partum periods on the meat fatty acids of suckling lambs. Meat Sci..

[69] Nudda A., Palmquist D.L., Battacone G., Fancellu S., Rassu S.P.G., Pulina G. (2008). Relationships between the contents of vaccenic acid, CLA and n–3 fatty acids of goat milk and the muscle of their suckling kids. Livest. Sci..

[70] Sanz-Sampelayo M., Fernández J., Ramos E., Hermoso R., Extremera F.G., Boza J. (2006). Effect of providing a polyunsaturated fatty acid-rich protected fat to lactating goats on growth and body composition of suckling goat kids. Anim. Sci..

